# Treatment strategy for reducing the risk of rituximab-induced cytokine release syndrome in patients with intravascular large B-cell lymphoma: a case report and review of the literature

**DOI:** 10.1186/1752-1947-7-280

**Published:** 2013-12-30

**Authors:** Katsuhiro Makino, Jumi Nakata, Satoru Kawachi, Tatsuyuki Hayashi, Atsuo Nakajima, Munehiro Yokoyama

**Affiliations:** 1 Department of General Medicine, Tokyo Metropolitan Police Hospital, 4-22-1 Nakano, Nakano-ku, Tokyo 164-8541, Japan; 2 Department of Hematology, Tokyo Metropolitan Police Hospital, 4-22-1 Nakano, Nakano-ku, Tokyo 164-8541, Japan; 3 Department of Diagnostic Pathology, Tokyo Metropolitan Police Hospital, 4-22-1 Nakano, Nakano-ku, Tokyo 164-8541, Japan

**Keywords:** Rituximab, CD20 antibody, Cytokine release syndrome, Systemic inflammatory response syndrome, R-CHOP, Diffuse large-cell lymphoma

## Abstract

**Introduction:**

Intravascular large B-cell lymphoma is a rare aggressive disseminated disease characterized by the presence of lymphoma cells in small vessels without lymphadenopathy. Rituximab, a novel monoclonal antibody against the CD20 B-cell antigen, has been reported to be effective in treating intravascular large B-cell lymphoma. However, adverse events have been reported in association with rituximab infusion.

**Case presentation:**

We report the case of a 54-year-old Japanese man diagnosed with Asian variant intravascular large B-cell lymphoma who died within five hours of the initiation of a first course of chemotherapy including rituximab. Autopsy results suggested that the patient died of severe systemic inflammatory response syndrome. A literature review revealed that rituximab administered during the second course of chemotherapy (instead of during the first course) appears to reduce the incidence of infusion reactions (from 48% to 15%) without altering the frequency of complete remission outcomes.

**Conclusions:**

Our data indicate that the incidence of adverse reactions to rituximab can be markedly decreased if the tumor load is first reduced with an initial course of chemotherapy excluding rituximab. Future prospective studies of the timing of rituximab administration are warranted.

## Introduction

Intravascular large B-cell lymphoma (IVLBCL) is a rare type of extranodal large B-cell lymphoma characterized by the proliferation of lymphoma cells within the lumina of small blood vessels and capillaries [1]. There is currently no standard therapeutic strategy for IVLBCL.

Many cases of IVLBCL have been successfully treated with a chemotherapy regimen comprising rituximab, cyclophosphamide, doxorubicin, vincristine, and prednisone (R-CHOP). Rituximab is a therapeutic monoclonal antibody against the CD20 B-cell antigen, and is reportedly effective in treating IVLBCL [2,3]. However, 84% to 95% of patients who receive a rituximab-based regimen experience treatment-related adverse events; approximately 90% of these are of mild to moderate severity, and most involve flu-like symptoms (fever, chills, nausea, and asthenia) [4]. The general overall risk of serious adverse events after rituximab administration is very low, but the compound has been associated with severe and unpredictable complications, including cytokine release syndrome (CRS), systemic inflammatory response syndrome (SIRS), and death [5,6]. Similar side effects have been reported during the treatment of other forms of cancer, such as acute lymphocytic leukemia and chronic lymphocytic leukemia [7,8]. The frequency of use of rituximab has been increasing as it is now applied to a myriad of conditions, and adverse events are more commonly reported [9]*.*

We here report a case of a patient diagnosed with Asian variant IVLBCL who died from SIRS during the first course of a chemotherapy regimen that included rituximab. We also present a review of the literature associated with rituximab use among patients with IVLBCL*.* To the best of our knowledge, this is the first such review. We observed that a simple alteration in the timing of rituximab infusion can markedly reduce the incidence of adverse effects without affecting treatment efficacy.

## Case presentation

A 54-year-old Japanese man with no relevant medical history presented at our hospital complaining of continuous fever and malaise of one month’s duration. His family history was unremarkable, and he had never smoked and did not frequently consume alcohol. Blood tests showed elevated levels of lactate dehydrogenase, soluble IL-2 receptor, and ferritin, as well as bicytopenia. A blood disorder, such as malignant lymphoma or hemophagocytic syndrome, was thus suspected. A bone marrow aspiration performed six days after admission revealed hemophagocytic syndrome, but without any evidence of malignancy. Accordingly, the patient underwent two sessions of steroid pulse therapy comprising intravenous administration of methylprednisolone sodium succinate (1000mg/day for three days) initiated on post-admission days 11 and 17. However, his general condition did not improve, and laboratory analysis revealed that his lactate dehydrogenase and soluble IL-2 receptor levels remained elevated, which was strongly suggestive of IVLBCL. Random skin biopsies of the abdominal wall, forearm, and thigh were all negative for IVLBCL. These areas were selected for ease of access. However, percutaneous liver biopsy revealed sinusoidal infiltration of lymphoma cells positive for CD20 and CD79a and negative for CD5. On the basis of these findings, the patient was diagnosed with Asian variant IVLBCL 22 days after admission.

The patient was treated with an R-CHOP-like chemotherapy regimen comprising rituximab (250mg/m^2^), cyclophosphamide (500mg/m^2^), doxorubicin (33mg/m^2^), and vincristine (0.9mg/m^2^) 24 days after admission. Prednisolone was not included because it had been administered since the diagnosis of hemophagocytic syndrome, and had been continuously administered during the 12 days before chemotherapy initiation. It is generally accepted that further prednisolone administration may have caused severe immunosuppression. Furthermore, in IVLBCL, rituximab-induced infusion reactions may occur more frequently than in other diseases because many tumor cells are lodged in blood vessels. We followed the guidelines of a Japanese IVLBCL study group, which recommended that all drug doses should be reduced by two-thirds to avoid tumor lysis syndrome after the first course of R-CHOP chemotherapy. The patient was administered cyclophosphamide, doxorubicin, and vincristine immediately before rituximab infusion, which was given at a calculated dose of 250mg/m^2^. Rituximab infusion was initiated at a rate of 25mg/h for the first hour along with acetaminophen and diphenhydramine. The infusion rate was gradually increased to 100mg/h by the second hour, at which point the patient complained of nausea. His temperature gradually increased to 41.2°C, and he had a rapid pulse of 126 beats/min. SIRS was diagnosed, and the infusion was discontinued. Arterial blood gas revealed a pH of 6.913 and high levels of lactic acid (16.0mmol/L; normal range: 0.5 to 1.8mmol/L), indicating lactic acidosis. Laboratory analysis revealed high levels of lactate dehydrogenase (2448IU/L; normal range 105 to 333IU/L), aspartate aminotransferase (635IU/L; normal range 10 to 34IU/L), alanine aminotransferase (640IU/L; normal range 10 to 40IU/L), and potassium (8.3mEq/L; normal range 3.5 to 4.5mEq/L). Unfortunately, we could not measure his serum IL-6 levels. Sustained ventricular tachycardia occurred due to metabolic derangement half an hour later. Hypotension became refractory to pressors, and the patient eventually died of asystole five hours after rituximab infusion.

Autopsy revealed invasion of tumor cells into the liver, lung, kidney, spleen, and pituitary gland. This case differs from another autopsy case in which the patient died of IVLBCL without undergoing chemotherapy. First, fewer tumor cells were present. Second, the tumor cells were too degenerated for immunohistochemical characterization (there was a loss of antigenicity). Finally, necrosis, degenerative atrophies, and ischemic changes were found in the cells and tissues of multiple organs. These findings suggest that SIRS accompanied the destruction of tumor cells by chemotherapy, which led to peripheral circulatory failure and ischemia in multiple organs. There was no evidence of active tumor lysis syndrome demonstrated by lower uric acid levels. Thus, the death of the patient was attributed to SIRS, which occurred subsequent to the rapid rituximab-induced destruction of tumor cells and the resulting cytokine release.

## Discussion

Intravascular large B-cell lymphoma is a rare variant of CD20^+^ large B-cell lymphoma, which is often suspected on the basis of clinical symptoms such as fever, night sweats, and weight loss, as well as on the basis of laboratory data such as elevated levels of serum lactate dehydrogenase and soluble IL-2 receptor [2,3]. These characteristic signs and symptoms have been postulated to be due in part to blood flow disturbance and microembolism as a result of the proliferation of lymphoma cells within the lumina of the small blood vessels, which leads to multiple organ damage [10].

Rituximab is a therapeutic monoclonal antibody against the B-cell marker CD20, and it has been shown to be effective in the treatment of IVLBCL [2,3]. The R-CHOP regimen comprises rituximab, cyclophosphamide, doxorubicin, vincristine, and prednisolone. The complete response (CR) rate is reportedly higher for patients with IVLBCL who have undergone chemotherapy with rituximab (R-chemotherapy group) compared with those who have not (chemotherapy group): in one study, a CR was achieved by 82% of patients in the R-chemotherapy group and by 51% of those in the chemotherapy group.

The overall risk of serious adverse events associated with the use of rituximab is very low*.* However, some patients have been reported to develop severe rituximab-induced infusion reactions, including CRS, SIRS, and death [5,6]. These noticeable adverse effects may be associated with a high tumor burden or advanced disease progression. The pathophysiology of this reaction is attributed to the release of cytokines, and usually occurs after the first administration of rituximab [5,8]. Massive cytokine release leads to the potentially severe condition known as CRS. The mechanism of cytokine release has been hypothesized to be related to the cross-linking of rituximab to CD20^+^ cells, subsequent complement activation and lysis of these target cells, and, finally, the release of target cell cytokines (such as IFN-γ, IL-6, IL-8, and TNF-α) within approximately two hours of the first antibody infusion [5,8]. Furthermore, macrophage recruitment contributes to the release of cytokines via interactions between macrophage Fcγ receptors and the Fc portion of the monoclonal antibody [11]. In addition, in severe cases of rituximab-induced CRS, a 5- to 10-fold increase in liver enzymes, elevation of D-dimer and lactate dehydrogenase levels, and prolongation of prothrombin time are commonly observed [12,13]. Cytokine release from apoptotic CD20^+^ tumor cells is more severe in patients with a higher tumor burden in their peripheral blood [12]. In such patients, CRS is prone to progress to SIRS. A cutoff value for cytokine levels in circulation has not been determined, and this phenomenon has been poorly studied. A gradual increase in the rituximab injection rate (for example, starting at 25mg/h and titrating higher at 25 to 50mg/h every 30min) may be useful for detecting CRS in its incipient stages and preventing SIRS.

To identify published data on IVLBCL treatments, we searched the Japan Medical Abstracts Society and PubMed websites using ‘intravascular’ and ‘lymphoma’ as search queries, and selected articles published between 1994 and 2011. The search was limited to IVLBCL patients. From these published clinical studies and case reports, as well as our own cases, we extracted cases in which rituximab had been used and checked the time of rituximab administration, its efficacy, and the incidence of adverse events. Using Fisher’s exact test, we evaluated the distribution of variables between the group that received rituximab during the first course (1st R-group) of chemotherapy and the group that received rituximab during the second or later courses (2nd R-group). All probability values were two-tailed and had an overall significance level >0.05.

The search of the literature published between 1994 and 2011 identified 56 clinical cases from 23 papers reporting the use of rituximab in patients diagnosed with IVLBCL. The data shown in Tables 1 and 2 indicate that the distribution of patients between the group that received rituximab during the first course and the group that received the drug during the second or later courses was equivalent [1,3,14-35]. The chemotherapy regimens employed comprised CHOP or modified CHOP. Adverse events related to rituximab infusion were observed in 18 (32%) of the 56 patients. Of these 18 patients, 14 (48%) were among the 29 included in the 1st R-group, and four (15%) were among the 27 included in the 2nd R-group. Patients who received rituximab during the first course of chemotherapy developed adverse events significantly more often than those who received the drug later in the treatment regimen (*P*=0.010). In the 1st R-group, five of these 14 patients developed hypoxia in association with the rituximab infusion, including Grade 3 severe hypoxia in two. Grade 3 sick sinus syndrome was also observed. All patients except for our present case recovered without complications (Table 1).

**Table 1:** Responses and adverse events

**Study/report**	**Timing of administration of rituximab**	**Response**	**Adverse events**
Shimada *et al*. [3]	1st: 23 cases	NA	10 of 23 cases developed infusion reactions; hypoxia was observed in 3 of the 10 cases and Grade 3 severe hypoxia in 1 case
Shimada *et al*. [3]	2nd: 25 cases	NA	4 of 25 cases developed infusion reactions
Ohkubo *et al*. [23]	1st	CR	None
Imahashi *et al*. [15]	1st	CR	NA
Aoyama *et al*. [14]	1st	NC	Grade 3 sick sinus syndrome
Kaku *et al*. [16]	1st	CR	None
Okachi *et al*. [24]	1st: 2 cases	CR	NA
Wakamatsu *et al*. [34]	1st	CR	NA
Shimizu *et al*. [27]	1st: 5 cases	CR	NA
Takahashi *et al*. [31]	1st	CR	NA
Tanikawa *et al*. [33]	1st	NC	NA
Sawa *et al*. [26]	1st	CR	NA
Watanabe *et al*. [35]	1st	CR	NA
Sakurai *et al*. [25]	1st	CR	NA
Takizawa *et al*. [32]	1st	CR	NA
Nakano *et al*. [22]	1st	NC	NA
Kotake *et al*. [21]	2nd	CR	NA
Shinoda *et al*. [28]	2nd	CR	NA
Shimada *et al*. [1]	2nd	CR	None
Ishizuka *et al*. [17]	2nd	CR	NA
Iwagami *et al*. [18]	2nd	CR	NA
Kashizaki *et al*. [19]	2nd	CR	Unspecified*
Kobayashi *et al*. [20]	2nd	PR	NA
Sakurai *et al*. [25]	2nd	CR	None
Suzuki *et al*. [29]	2nd	CR	NA
Tadokoro *et al*. [30]	2nd	CR	NA
Our other case	1st	CR	Grade 1 fever, chills, hypoxia
Present case	1st	NA	Death

NA, not applicable; CR, complete response; NC, no change; PR, partial response. *In this case, rituximab administration was attempted during the first course, but an infusion reaction was observed and the rituximab injection was discontinued. However, rituximab was administered according to protocol during the second course.

**Table 2:** Adverse events related to rituximab administration

**Timing of rituximab administration**	**Number of patients who suffered from infusion reactions**	**Number of patients who had no infusion reactions**	**Total number of patients**
With the first course of chemotherapy	14	15	29
With or after the second course of chemotherapy	4	23	27

Furthermore, the data shown in Tables 1 and 3 indicate that a CR was achieved by 17 (85%) of 20 patients in the 1st R-group and nine (90%) of 10 patients in the 2nd R-group. There was no significant difference between the groups in terms of the CR rate (*P*=1.000). Our literature review suggests that treatment regimens introducing rituximab during the second or later courses of chemotherapy are associated with lower risks of adverse effects without affecting treatment efficacy.

**Table 3:** Efficacy of chemotherapy

**Timing of rituximab administration**	**Number of patients who achieved a complete response**	**Number of patients who did not achieve a complete response**	**Total number of patients**
With the first course of chemotherapy	17	3	20
With or after the second course of chemotherapy	9	1	10

Thus, initiating rituximab administration during the second course of chemotherapy appears to reduce the incidence of infusion reactions without changing the CR rate. This is in agreement with a clinical study that reported that post-CHOP administration of rituximab significantly reduced the incidence of adverse reactions compared with pre-CHOP administration [36]. The most probable mechanism is the reduction of tumor cells during the first course of chemotherapy via a regimen such as CHOP. It is very important to prevent CRS in patients with IVLBCL since IVBCL is associated with hemophagocytic syndrome and thus elevated cytokine levels. In patients with chronic lymphocytic leukemia, large amounts of white blood cells (>50.0×10^3^ cells/μL) are a risk factor for rituximab-induced infusion reactions [12,13]. However, there is no way to measure the amount of tumor cells present in patients with IVLBCL, although it has been reported that patients with splenomegaly or tumor cells detectable in the peripheral blood vessels tend to experience infusion reactions [12,13]. Considering these reports, a simple change in the timing of rituximab infusion appears to markedly reduce the incidence of adverse effects without affecting treatment success rates. Certainly, however, these data are from a small number of patients, and the suggestion needs to be verified by prospective randomized controlled trials.

It has also been reported that patients who have not undergone steroid therapy and/or chemotherapy before receiving the first dose of rituximab show a tendency to develop adverse events related to its infusion, more so than those who have undergone these treatments beforehand. We did not use steroids immediately before the injection of rituximab in the present case, but it could have been helpful in preventing an infusion reaction.

## Conclusions

This case report demonstrates that rituximab administration during the first course of chemotherapy can be extremely dangerous for IVLBCL patients. Our literature review indicates that the incidence of adverse reactions can be markedly decreased if the tumor load is first reduced with an initial course of rituximab-free chemotherapy followed by second or later courses of a rituximab-based regimen. However, these data have been derived from a small number of patients, and future prospective studies of the timing of rituximab administration are warranted.

## Consent

Written informed consent was obtained from the patient’s next of kin for publication of this case report and any accompanying images. A copy of the written consent is available for review by the Editor-in-Chief of this journal.

## Abbreviations

CR: Complete response; CRS: Cytokine release syndrome; Fc: Crystallizable fragment (of an antibody); IFN: Interferon; IL: Interleukin; IVLBCL: Intravascular large B-cell lymphoma; SIRS: Systemic inflammatory response syndrome; TNF: Tumor necrosis factor.

## Competing interests

The authors declare that they have no competing interests.

## Authors’ contributions

KM, JN, and SK followed up the patient, collected the data, reviewed the literature, collected all the information, and wrote the manuscript. TH and AN contributed to patient management and data collection. MY participated by providing pathological reports. All authors read and approved the final version of the manuscript.
